# A Synthetic Virus-Like Particle Streptococcal Vaccine Candidate Using B-Cell Epitopes from the Proline-Rich Region of Pneumococcal Surface Protein A

**DOI:** 10.3390/vaccines3040850

**Published:** 2015-10-16

**Authors:** Marco Tamborrini, Nina Geib, Aniebrys Marrero-Nodarse, Maja Jud, Julia Hauser, Celestine Aho, Araceli Lamelas, Armando Zuniga, Gerd Pluschke, Arin Ghasparian, John A. Robinson

**Affiliations:** 1 Swiss Tropical and Public Health Institute, Socinstrasse 57, Basel 4051, Switzerland; E-Mails: marco.tamborrini@unibas.ch (M.T.); maja.jud@gmx.net (M.J.); julia.hauser@unibas.ch (J.H.); Celestine.Aho@cdu.edu.au (C.A.); araceli.lamelas@unibas.ch (A.L.); Gerd.Pluschke@unibas.ch (G.P.); 2 University of Basel, Petersplatz 1, Basel 4003, Switzerland; 3 Virometix AG, Wagistrasse 14, Schlieren 8952, Switzerland; E-Mails: n.geib@web.de (N.G.); aniebrys.marrero@chem.uzh.ch (A.M.-N.); armando.zuniga@virometix.com (A.Z.); 4 Department of Chemistry, University of Zürich, Winterthurerstrasse 190, Zürich 8057, Switzerland

**Keywords:** peptide, streptococcus, synthetic virus-like particle, PspA, vaccine

## Abstract

Alternatives to the well-established capsular polysaccharide-based vaccines against *Streptococcus pneumoniae* that circumvent limitations arising from limited serotype coverage and the emergence of resistance due to capsule switching (serotype replacement) are being widely pursued. Much attention is now focused on the development of recombinant subunit vaccines based on highly conserved pneumococcal surface proteins and virulence factors. A further step might involve focusing the host humoral immune response onto protective protein epitopes using as immunogens structurally optimized epitope mimetics. One approach to deliver such epitope mimetics to the immune system is through the use of synthetic virus-like particles (SVLPs). SVLPs are made from synthetic coiled-coil lipopeptides that are designed to spontaneously self-assemble into 20–30 nm diameter nanoparticles in aqueous buffer. Multivalent display of epitope mimetics on the surface of SVLPs generates highly immunogenic nanoparticles that elicit strong epitope-specific humoral immune responses without the need for external adjuvants. Here, we set out to demonstrate that this approach can yield vaccine candidates able to elicit a protective immune response, using epitopes derived from the proline-rich region of pneumococcal surface protein A (PspA). These streptococcal SVLP-based vaccine candidates are shown to elicit strong humoral immune responses in mice. Following active immunization and challenge with lethal doses of streptococcus, SVLP-based immunogens are able to elicit significant protection in mice. Furthermore, a mimetic-specific monoclonal antibody is shown to mediate partial protection upon passive immunization. The results show that SVLPs combined with synthetic epitope mimetics may have potential for the development of an effective vaccine against *Streptococcus pneumoniae*.

## 1. Introduction

*Streptococcus pneumoniae* is a major cause of disease in humans including severe meningitis, otitis media, pneumonia, and sepsis [1,2]. Millions of individuals die of diseases caused by *S. pneumoniae* every year, and most of these deaths occur in developing countries [3]. Pathogenic pneumococci produce structurally and antigenically diverse polysaccharide capsules that can be used to identify more than 90 immunochemically distinct serotypes. The isolated capsular pneumococcal polysaccharides (CPPs) have been used for many years as vaccines to confer serotype-specific, antibody-mediated protection against invasive pneumococcal disease. However, CPPs alone are typically poorly immunogenic, elicit mostly IgM-type antibodies, induce weak protection in children and fail to elicit long-lasting memory responses in adults [4,5]. Second-generation pneumococcal vaccines composed of isolated capsular polysaccharide conjugated to carrier proteins (PCPs) result in improved T-cell dependent immune responses [6,7]. Since their introduction in the 1990s, marketed PCP vaccines have proven highly effective in combating invasive pneumococcal disease [8,9]. However, their production requires a complex manufacturing process [10], they can be poorly thermostable [11] and may confer only poor or variable levels of protection in some population groups [12] and against some disease states, including total pneumococcal meningitis and total pneumococcal otitis media [4]. Moreover, PCP vaccines offer only limited serotype coverage and their use promotes serotype replacement and emergence of highly virulent capsule switch variants expressing altered capsule polysaccharides not covered by the vaccine [13,14,15]. Consequently, there is a pressing need for the development of alternative serotype-independent pneumococcal vaccines.

One promising approach focuses on the development of recombinant subunit vaccines using highly conserved pneumococcal surface proteins and virulence factors [4,5,16,17], which can be administered together with an immunostimulatory adjuvant [16,17]. Difficulties can arise, however, when the recombinant proteins are unstable or incorrectly folded and/or direct immune responses towards immunodominant polymorphic epitopes irrelevant for protection, while avoiding immune responses against neutralizing conserved epitopes [18].

**Figure 1:**
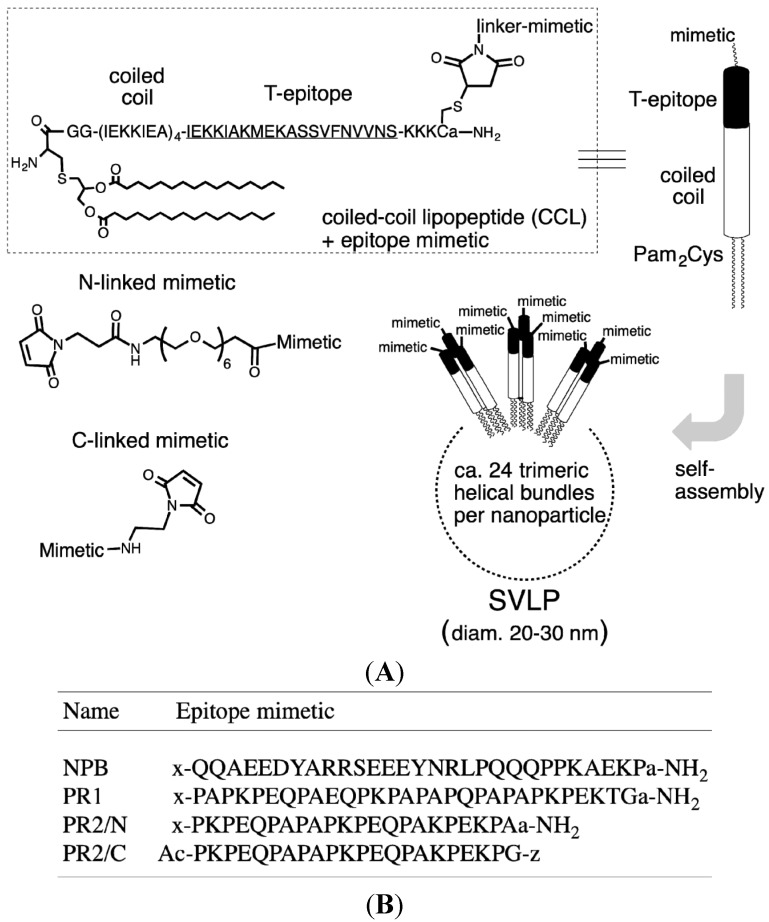
Components and their self-assembly into SVLPs. (**A**), SVLPs are composed of a synthetic coiled-coil lipopeptide (CCL), which contains a heptad repeat (IEKKIEA)_4_ linked to a promiscuous T helper epitope (underlined) and to the TLR-2/6 ligand Pam_2_Cys. B-cell epitope mimetics can be conjugated to the unique Cys, as shown. The C-terminal a-NH_2_ is D-Ala-amide to protect against digestion with carboxypeptidases. The CCLs form trimeric helical bundles that self-assemble into 20–30 nm diameter nanoparticles (SVLPs) with a lipid-chain core; (**B**) In this work, PRR-derived peptides were linked through either the N- or C-terminus (represented in the sequences by x and z, respectively) to the unique Cys of the CCLs using the linkers shown in (A).

We explore here an alternative approach, using synthetic epitope mimetics as antigens to focus immune responses onto conserved protective B-cell epitopes. The mimetics are delivered to the immune system using synthetic virus like particles (SVLPs). SVLPs arise from synthetic coiled-coil lipopeptides (CCLs) that are designed to spontaneously self-assemble into nanoparticles in aqueous buffer. The coiled coil directs formation of parallel trimeric helical bundles, and the lipid tail in each CCL drives formation of 20–30 nm diameter nanoparticles, through association of around 24 trimeric helical bundles and burial of the lipid chains in a central micelle-like core. A B-cell epitope mimetic conjugated to each CCL can then be displayed in a multivalent format (*ca.* 70 copies) over the outer surface of the nanoparticle (Figure 1A) [19,20,21]. In addition, each CCL is designed to include selected T cell epitopes along with a toll-like receptor ligand, such as the lipids Pam_2/3_-Cys [20,22]. The inclusion of T-cell epitopes and ligands for pattern recognition receptors should aid the tailored activation of both innate and adaptive immunity. An additional feature is that the manufacturing process for SVLP vaccines is based entirely on synthetic chemistry, which gives products of well-defined composition, high purity and thermal stability, and no external adjuvant is used. This is in contrast to VLPs based on viral capsid proteins in established vaccines targeting hepatitis B and E, as well as human papilloma virus, which require challenging cell-based methods for production and purification, and administration with an alum adjuvant to boost immunogenicity [23,24,25].

**Figure 2:**
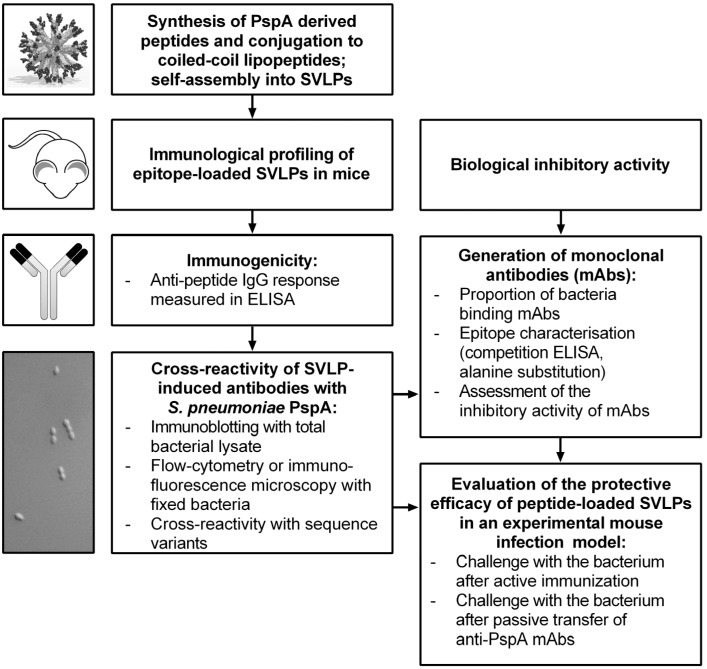
Testing of a potential SVLP-based pneumococcal vaccine candidate. Designed coiled-coil lipopeptides carrying segments of PspA were made by chemical synthesis (top, left). Laboratory mice were immunized with the resulting SVLPs. The murine immunological responses to the SVLPs were analyzed to determine *S. pneumoniae* cross-reactive antibody responses and to generate mAbs. Challenge experiments were used to evaluate protective humoral immune responses.

In this work, epitope mimetics based on pneumococcal surface protein A (PspA), displayed on the surface of the SVLPs, have been tested as vaccine candidates for their ability to provide protection against pneumococcal infection. An overview of the work-flow undertaken is given in Figure 2.

PspA is a well-studied subunit pneumococcal vaccine candidate [16]. During invasive systemic infection, PspA interferes with the deposition of complement on the pneumococcal surface, and during mucosal infection PspA protects the bacteria from killing by human lactoferrin [26,27,28,29]. PspA contains a variable α-helical N-terminal domain, an antigenically conserved proline-rich region (PRR), which is often interrupted by a non-proline block (NPB), and a choline-binding domain that anchors the protein to the bacterial outer cell wall [30,31]. Antibodies to PspA are capable of protecting mice against lethal infection with *S. pneumoniae* (reviewed in [16]) and protective B-cell epitopes have been found within the polymorphic N-terminal domain as well as in the PRR [31,32,33]. In particular, antibodies specific for the semi-conserved PRR have been found to provide cross-protection against a broad spectrum of pneumococcal strains. This work explores the properties of SVLPs assembled from CCLs carrying B-cell epitope mimetics from the PRR of PspA. These SVLPs are shown to be highly immunogenic in mice without additional adjuvants, where they elicit *S. pneumoniae* cross-reactive antibodies and generate significant protective humoral immune responses.

## 2. Methods and Materials

### 2.1. Synthesis of Peptides and Coiled-Coil Lipopeptides

The CCL used in this work is shown in Figure 1A, and was synthesized as described earlier [20]. The epitope mimetics studied are linear peptides from the PRR or NPB of PspA (shown in Figure 1B). The linear sequences were synthesized by Fmoc-solid-phase peptide synthesis [34], using either Rink amide MBHA resin (for C-terminal amides) or chlorotrityl chloride resin (for peptides with a carboxylic acid terminus) (Novabiochem, Merck-Millipore, Darmstadt). For N-linked mimetics (NPB, PR1 and PR2/N), Fmoc-21-amino-4,7,10,13,16,19-hexaoxaheneicosanic acid (Fmoc-NH-PEG_6_-COOH, Novabiochem, Merck-Millipore, Darmstadt, Germany) as linker was coupled to the N-terminus of the resin-bound peptide. The Fmoc group was then removed with 20% piperidine in DMF and 3-maleimidopropionic acid (Sigma-Aldrich, St Louis, MO, USA) was coupled to the new N-terminus. After cleavage from the resin and side-chain deprotection under standard conditions [34], the products were purified by HPLC (C18 column, 10%–40% MeCN in H_2_O (+0.1% CF_3_COOH). Each product was >95% pure by analytical HPLC. Analysis by MALDI-TOF-MS: PR1, *m*/*z* [M + H]^+^ 3609.7, calc. 3609.1; PR2/N, *m*/*z* [M + H]^+^ 2888.8, calc. 2889.3; NBP *m*/*z* [M + H]^+^ 4071.0, calc. 4072.3.

For the C-terminal conjugate (PR2/C), the assembled peptide chain was acetylated at the N-terminus before cleavage from the resin with CF_3_CH_2_OH/CH_2_Cl_2_ (1:4). After concentration *in vacuo*, the side-chain protected peptide was precipitated with cold Et_2_O, washed and dried. (2-Aminoethyl)maleimide (Sigma-Aldrich, St. Louis, MO, USA) was coupled to the C-terminus in DMF using HATU/HOAt/*i*Pr_2_EtN. After concentration *in vacuo* and precipitation with Et_2_O, the peptide was washed and dried. The side chain protecting groups were then removed under standard conditions [34], and the product was purified by HPLC (C18 column, 10%–100% MeCN in H_2_O (+0.1% CF_3_COOH); >95% pure by analytical HPLC. Analysis by ES-MS: *m*/*z* [M + H]^+^ 2483.2, calc. 2484.8.

For conjugation of each epitope mimetic to the CCL, the CCL (6.0 mg, 0.9 µmol) and epitope mimetic (1.3 µmol) in H_2_O/MeCN (1:1, 5 ml) at pH 6.5–7.0, was stirred for 2 h at room temp. The conjugate was then purified by HPLC using a C4 preparative column with 50%–100% MeCN in H_2_O (+0.1% TFA); each conjugate purity was >95%. For CCL-PR1, MALDI-TOF MS, *m/z* [M + H] 10406.8, calc. 10405.6. For CCL-NPB, MALDI-TOF MS, *m/z* [M +H] 10869.8 Da, found 10872.3. For CCL-PR2/N, MALDI-TOF MS *m/z* [M + H] 9686.2, calc. 9685.6; for CCL-PR2/C, MALDI-TOF MS, *m/z* [M + H] 9280.2, calc. 9280.2.

The conjugates in phosphate buffered saline (PBS) were analyzed by dynamic light scattering (Wyatt DynaPro, Wyatt Technologies, Santa Barbara, CA, USA) at 25 °C. The mean hydrodynamic radius (R_h_ in nm) and polydispersity (%Pd) were 12 nm and 12.3% for CCL-PR1; 12 nm and 13.0% for CCL-PR2/N; 11 nm and 16% for PR2/C; 14 nm and 13% for CCL-NPB.

Each epitope-CCL conjugate was dissolved in sterile PBS and equilibrated for 40 min at room temperature with occasional mixing, prior to immunizations.

### 2.2. Cloning and Expression of the PRR from PspA Strain 1577 (rPspA)

An internal 312 bp fragment of *pspA* from *S. pneumoniae* strain 1577 was amplified by PCR using the following primers, cloned between *Nco* I and *Sal* I restriction sites in pET32a (*Novagen*) and expressed as a thioredoxin (Trx) fusion protein (called rPspA), as described earlier [31]:

PspA-for: 5'-GGGAG*CCATGG*CTGACCTTAAGAAAGCAGTTAATGAGCCA-3'

PspA-rev: 5'-CC*GTCGAC*ACTACATACCGTTTTCTTGTTTCCAGCC-3'

**Figure A1:**
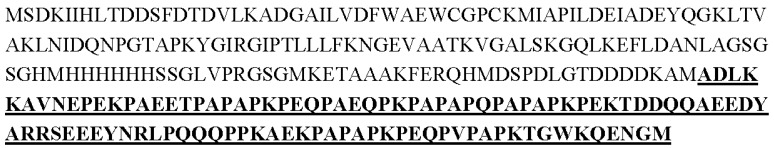
The amino acid sequence of the fusion protein (rPspA), comprising the PRR of PspA linked to thioredoxin (Trx). The PRR is bold and underlined.

The plasmid was introduced into *E. coli* BL21(DE3) and rPspA was expressed in LB medium. The full amino acid sequence of the fusion protein is given in the Appendix (Figure A1). rPspA expression was induced at OD_600_ 0.6–0.9 by addition of IPTG (0.1 mM). Induced cells (3 h at 37 °C) were harvested and lysed by sonication in 20 mM Tris pH 8, 500 mM NaCl, 10 mM imidazole (buffer A) containing a protease inhibitor cocktail (Roche, Basel, Switzerland) and DNAse. Lysate was loaded onto a Ni-NTA 1 mL Superflow cartridge (Qiagen, Venlo, The Netherland), non-bound proteins washed out with buffer A and rPspA eluted with buffer containing 300 mM imidazole, purified by size-exclusion chromatography (Superdex 200 10/300 GL) in 20 mM Tris pH 7.5, 150 mM NaCl. SDS-PAGE revealed a single band with an apparent molecular weight of 30 kDa. For immunizations, rPspA was concentrated and filtered using a 0.2 µm filter, dialyzed into Hanks’ Balanced Salt solution (HBSS) and adsorbed to alum (Imject Alum, Pierce Biotechnology, Rockford, IL, USA). Briefly, Imject alum was added dropwise with constant mixing to the rPspA solution. Mixing was continued for 30 min to allow alum to effectively adsorb antigen. The final volume ratio of Imject alum to protein solution was 1:1.

### 2.3. Pneumococcal Strains

Clinical isolates of *S. pneumoniae* (Table 1) were collected during surveillance studies in Ghana or in Papua New Guinea (PNG). Isolates from PNG were obtained from the Papua New Guinea Institute of Medical Research (IMR), Goroka. *S. pneumoniae* colonies were identified on the basis of colony morphology. All pneumococcal isolates were serotyped by a sequential multiplex PCR as described [35]. The DNA sequence of pspA in each strain was determined using the primers given in the Appendix Table A1.

**Table 1:** Cross-reactivity of immune-sera with genetically diverse pneumococcal clinical isolates in flow-cytometric analyses. Capsular serotypes and pspA allelic variants that have been classified into clades according to [30] are shown. (+ = surface staining; − = no surface staining). A maximum likelihood phylogenetic tree of the PRR sequences of the 10 tested *S. pneumoniae* clinical isolates and of 44 *S. pneumoniae* genomes from the NCBI database is shown in the Appendix (Figure A2). CSF = Cerebrospinal fluid.

Strain	Diagnosis of Patient	Site of Isolation	Origin	Year Isolated	*pspA* Clade	Capsule Type	Non-Proline Block	Cross-Reactivity of Sera Induced by Immunogen			
								CCL-NPB	CCL-PR1	CCL-PR2/N	CCL-PR2/C
1577	case	CSF	Ghana	2009	1	1	present	+	+	+	+
233	case	CSF	PNG, Chimbu	1997	1	4	present	+	+	+	+
716	case	CSF	PNG, Lufa	1999	1	5	present	+	+	−	−
920	carriage	Naso-pharynx	PNG, Eastern Highlands	2006	1	8	present	+	+	+	+
1388	case	CSF	PNG, Lufa	2002	2	4	present	+	+	+	+
932	case	CSF	PNG, Goroka	1999	3	6A/B	absent	−	+	+	+
839	case	CSF	PNG, Ung/Bena	1999	3	7F	absent	−	−	+	+
1260	case	CSF	PNG, Goroka	2001	4	4	present	+	+	−	−
1272	case	CSF	PNG, Daulo	2002	5	2	present	+	+	+	+
4408	carriage	Naso-pharynx	PNG, Eastern Highlands	2010	5	19A	present	+	+	+	+

### 2.4. Animal Studies

Animal experiments were carried out in accordance with the national rules and regulations for the protection of animal rights. The protocols were ethically approved by the veterinary office of the county of Basel-City, Switzerland.

Six to eight week-old specific pathogen-free HsdWin:NMRI outbred mice were purchased from Harlan Laboratories B.V. (The Netherlands) and used for immunizations and for pneumococcal protection studies. For immunogenicity studies, mice received two subcutaneous injections (100 µL) of 20 µg of recombinant PspA formulated with Imject alum, or SVLPs in PBS without adjuvant, at three-week intervals. The used SVLP immunization dose was selected based upon preliminary dose-finding studies in NMRI mice. Blood was collected before the first injection and 10 days after the final injection. Negative controls received the same course of sterile PBS.

The protective efficacy of SVLPs was evaluated in an experimental mouse model of acute infection in which mice were challenged intravenously (i.v.) via the tail vein with a lethal dose (approximately 10^4^ CFUs) of a serotype 1 clinical isolate (1577). Pneumococci recovered from the blood of intraperitoneally infected mice were used to prepare bacterial stock cultures. The mouse-passaged pneumococcal strain was grown overnight on blood agar plates (Columbia agar with sheep blood, *Oxoid* GmbH) at 37 °C in 5% CO_2_. Pneumococci were harvested with a loop, resuspended in sterile HBSS and adjusted to 1 × 10^5^ CFUs/mL before administration. After challenge the animals were monitored for 14 days for signs of illness. Mice were euthanized when they exhibited ruffled fur, hunched back, paralysis or other symptoms. Mouse survival curves were evaluated statistically by the log rank (Mantel-Cox) test using GraphPad Prism Software (version 6.0 for Windows, GraphPad Software, Inc., La Jolla, CA, USA). A P value of ≤0.05 was considered statistically significant.

### 2.5. Generation of Hybridoma Cell Lines Producing Epitope Mimetic-Specific mAbs

Cells from the spleen of a CCL-PR2/N (Figure 1B) immunized mouse were fused with PAI mouse myeloma cells as a fusion partner. Hybrids were selected in hypoxanthine-aminopterin-thymidine (HAT) medium, and B-cell hybridoma lines secreting peptide-specific IgG were identified by peptide ELISA. For large-scale mAb production, cloned hybridoma cell lines were cultured in 175 cm^2^ flasks and mAbs were purified by protein A affinity chromatography (HiTrap rProtein A FF, Amersham Biosciences AB, Uppsala, Sweden). Purified mAbs were dialyzed against PBS, sterile-filtered, and stored at −80 °C.

### 2.6. Enzyme-Linked Immunosorbent Assay (ELISA)

ELISA MaxiSorp microtitre plates (Thermo Fisher Scientific, Nunc A/S, Roskilde, Denmark) were coated at 4 °C overnight with peptide in PBS (50 µL; 5 µg/mL). Wells were blocked with 5% milk powder in PBS for 1 h at room temperature and washed 3 × with PBS containing 0.05% Tween 20. Plates were then incubated with serial dilutions of mouse sera in PBS for 1 h at room temperature. After washing, plates were incubated with alkaline phosphatase-conjugated goat anti-mouse IgG (γ-chain-specific) antibodies (Sigma, St. Louis, MO, USA) for 1 h at room temperature and then washed. Phosphatase substrate (1 mg/mL p-nitrophenyl phosphate) in buffer (0.14% Na_2_CO_3_, 0.3% NaHCO_3_, 0.02% MgCl_2_) was added and incubated at room temp. The optical density (OD) of the reaction product was recorded after an appropriate time at 405 nm using a microplate reader (Sunrise, Tecan Trading AG, Männedorf, Switzerland). Endpoint titer is the last serum dilution where the OD_test serum_ ≥ mean OD_PBS_ plus two times the standard deviation. In competition assays, immune-sera or mAbs were pre-incubated with increasing concentrations of competitor peptides in PBS for 30 min. Equilibrated antibody-peptide solutions were transferred to peptide-coated ELISA plates and processed as described above.

### 2.7. SDS-PAGE and Immunoblotting

*S. pneumoniae* isolates were grown overnight on blood agar plates, collected and washed once in sterile PBS. Pelleted bacteria (4.25 × 10^7^ CFUs) were lysed in 100 μL sample buffer (Laemmli buffer, Bio-Rad Laboratories, Inc. Hercules, CA, USA) and heated to 95 °C for 10 min. before loading on 4%–12% Bis-Tris gels. Following gel electrophoresis, separated proteins were electrophoretically transferred to a nitrocellulose membrane. Blots were blocked with PBS containing 5% milk powder for 1 h at room temperature or overnight at 4 °C. The membrane was incubated with appropriate dilutions of mouse serum or mAbs in PBS for 1 h at room temperature. In competition experiments, primary antibodies were pre-incubated for 30 min. with a competitor peptide at a concentration of 1, 0.1 or 0.01 μg/mL. After several washing steps, filter strips were incubated with goat anti-mouse IgG horseradish peroxidase conjugated Ig (0.1 μg/mL; Bio-Rad Laboratories, Inc. Hercules, CA, USA) for 1 h. Blots were developed using the ECL (*Pierce*) system according to manufacturer’s instructions.

### 2.8. Surface Staining of Bacterial Cells

*S. pneumoniae* isolates were grown overnight on blood agar plates or in liquid medium (Bacto Todd Hewitt Broth supplemented with Bacto yeast extract), collected and resuspended in sterile PBS. For flow cytometry experiments, pelleted bacteria were inactivated in formalin (1 mL) for 30 min at room temperature and blocked for 30 min at 4 °C with blocking solution containing fatty acid-free bovine serum albumin (BSA) in PBS (5 mg/mL). Fixed bacteria (7.2 × 10^5^ CFUs per sample) were stained with mouse immune-sera or mAbs for 1 h at room temperature. Surface bound IgG was detected with Alexa-Fluor 488-conjugated goat anti-mouse IgG antibodies (Invitrogen). Cell pellets were resuspended in 400 µL FACS flow and bacterial staining was analysed by using a FACS Calibur cytometer (Becton Dickinson, Hongkong, China).

For immunofluorescence microscopy, 30 µL droplets of formalin solution were placed into each well of a pre-coated Poly-L-Lysin microscope glass slide. Ten microliters of a bacterial suspension containing approximately 2.6 × 10^8^ CFUs were added to each well and incubated for 30 min at room temp. After washing, wells were blocked with 1% BSA in PBS. Immunostaining was performed by incubating the wells with an appropriate mAb or serum dilution in blocking solution for 1 h at room temperature. After washing, cells were incubated with secondary antibodies specific for mouse IgG conjugated with Alexa-Fluor 488. Before the immunoreactivity was analyzed with a fluorescence microscope, the wells were washed, mounted with ProLong^®^ Gold antifade reagent (Invitrogen) and covered with a coverslip.

## 3. Results

### 3.1. Design and Synthesis of Epitope-Loaded SVLPs

Protective epitopes have been identified within the PRR of PspA [31,36]. For our work, two epitopes (PR1 and NPB) were selected from the PRR of a serotype 1 pneumococcal strain (1577) (Figure 1B). A second epitope (PR2) derived from a heterologous PRR was also selected. For the generation of epitope-loaded SVLPs, linear peptides representing the PR1 and NPB epitopes were each coupled through the N-terminus to the CCL building block (Figure 1A). The conjugates are denoted CCL-PR1 and CCL-NPB (Figure 1B). The PR2 peptide was coupled via a linker to the CCL in either N- or C-terminal orientations, yielding two additional constructs (CCL-PR2/N and CCL-PR2/C, see Figure 1B). The CCL building block, described in earlier work [20], contains the isoleucine zipper (IEKKIEA)_4_, which directs formation of thermally stable parallel trimeric helical bundles [37]. The isoleucine zipper is fused to a CD4 T helper epitope. In this case, a promiscuous T helper epitope was chosen, derived from the circumsporozoite surface protein of the malaria parasite *Plasmodium falciparum* (Figure 1A), since this epitope is recognized by T cells in association with most mouse and human MHC class II molecules [38,39]. The CCL is therefore expected to elicit T helper responses in the mouse infection model used in this work, as well as in humans. As in earlier studies [19,20,21,22], self-assembly of the CCL-epitope conjugates into SVLPs was confirmed by dynamic light scattering measurements, which showed narrow nanoparticle size distributions with mean hydrodynamic radii (R_h_) in the range of 12–14 nm and polydispersity (%Pd) values between 12.3% and 16%.

### 3.2. Immunogenicity of Epitope-Loaded SVLPs in Mice

**Figure 3:**
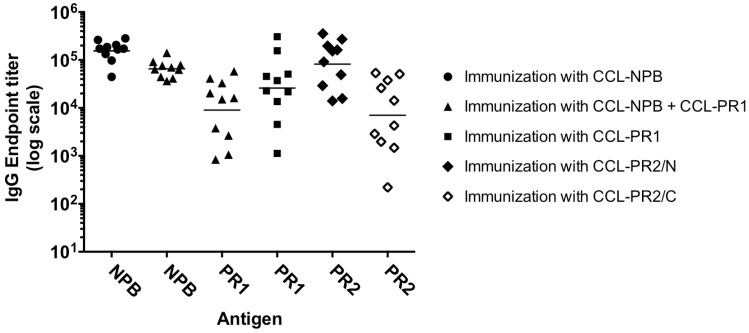
Anti-peptide IgG response in NMRI mice. Female NMRI mice (6–8 weeks of age) were immunized twice with SVLP constructs: CCL-NPB, CCL-PR1, CCL-NPB + CCL-PR1 (mixture), CCL-PR2/N or CCL-PR2/C (see Figure 1B). ELISA endpoint IgG titers measured against the target peptide (Antigen) for individual animals (symbol) and the geometric mean (black line) after the second immunization are shown. Preimmune sera showed no reaction in ELISA.

NMRI outbred mice (10 per group) were immunized twice with 20 µg doses of each SVLP construct: CCL-PR1, CCL-NPB, CCL-PR2/N, CCL-PR2/C or a mixture of CCL-NPB + CCL-PR1, each in PBS without adjuvant. ELISA of sera collected pre-immune and after the second immunization showed that most mice developed strong antigen-specific IgG responses against the target peptide (Figure 3). SVLPs comprising a mixture of CCL-NPB and CCL-PR1 elicited high antibody titers against both the NPB and the PR1 epitopes. The CCL-PR2/C construct elicited lower titers of PR2-specific IgG antibodies compared to CCL-PR2/N (*p* = 0.0065). Pre-immune sera showed no reactivity in ELISA.

### 3.3. Cross-Reactivity of Induced Antibodies with *S. Pneumoniae* PspA

**Figure 4:**
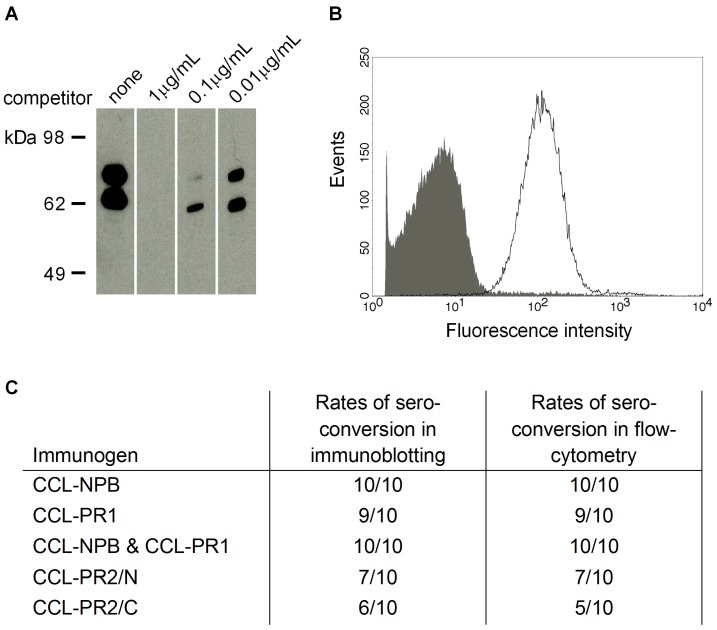
Cross-reactivity of sera raised against peptide-loaded SVLPs with *S. pneumoniae*. (**A**), competition Western blot analysis with total bacterial lysate of the *S. pneumoniae* 1577 clinical isolate and the PR2/N synthetic peptide. Total lysates were separated by SDS-PAGE under reducing conditions and blotted onto a nitrocellulose membrane. Serum of a NMRI mouse immunized with CCL-PR2/N was pre-incubated with or without peptide competitor and subsequently added to cut strips. Immune serum was used at a dilution of 1:2000 and the competitor at a concentration of 1, 0.1 or 0.01 μg/mL; (**B**), flow cytometry-based surface staining was performed with formalin-inactivated pneumococci (1577) using CCL-PR2/N specific mouse serum and fluorescently labeled secondary antibodies. The signal resulting from staining with pre-immune serum is shown as gray area; PspA specific signals are shown with a black line. Sera were used at a dilution of 1:100; (**C**), Rates of sero-conversion for immunoblotting and flow-cytometry analyses with sera from immunized mice taken after the second immunization with peptide-loaded SVLPs.

Cross-reactivity of serum IgGs raised against the peptide-loaded SVLPs with the endogenous protein expressed by *S. pneumoniae* was investigated by immunoblotting and flow-cytometry analysis. The molecular mass of PspA ranges between 67 kDa and 98 kDa in various pneumococcal strains [40]. With the *S. pneumoniae* 1577 clinical isolate, immune sera stained a double band with an apparent molecular weight of around ~70 kDa. In Western blot competition experiments, the binding of immune sera to the endogenous protein was inhibited by the corresponding synthetic peptide in a concentration dependent manner (Figure 4A), thereby confirming the specificity of the binding. Flow-cytometry analyses with formalin-inactivated bacteria showed the development of surface-binding IgG antibodies in sera from immunized mice. Surface staining of cells with PspA specific antibodies was assessed using bacteria grown on blood agar plates as well as bacteria harvested from liquid cultures. Both preparations yielded similar results in flow cytometry experiments. Representative results are shown with serum of a CCL-PR2/N immunized mouse in Figure 4B. While all mice receiving CCL-NPB alone or in combination with CCL-PR1 sero-converted in immunoblotting and flow-cytometry analyses, lower rates of sero-conversion were observed after immunization with CCL-PR1, CCL-PR2/N or CCL-PR2/C (Figure 4C). None of the pre-immune mouse sera was positive in flow-cytometry or Western blot analysis.

### 3.4. Cross-Reactivity with Strains Expressing Heterologous PspA

**Figure A3:**
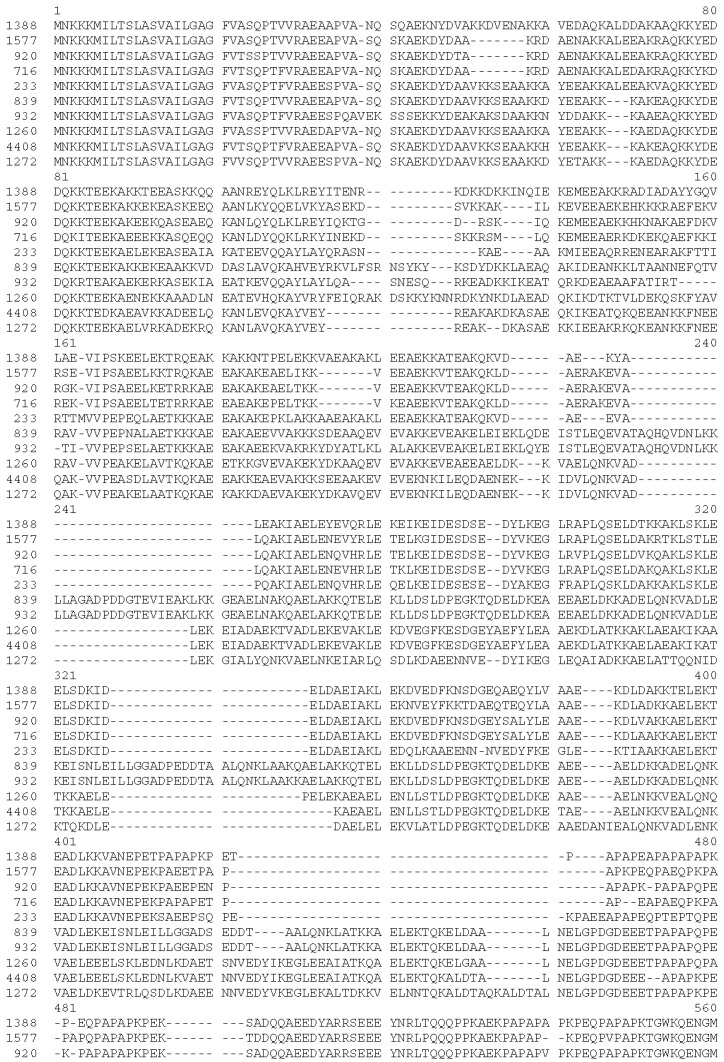

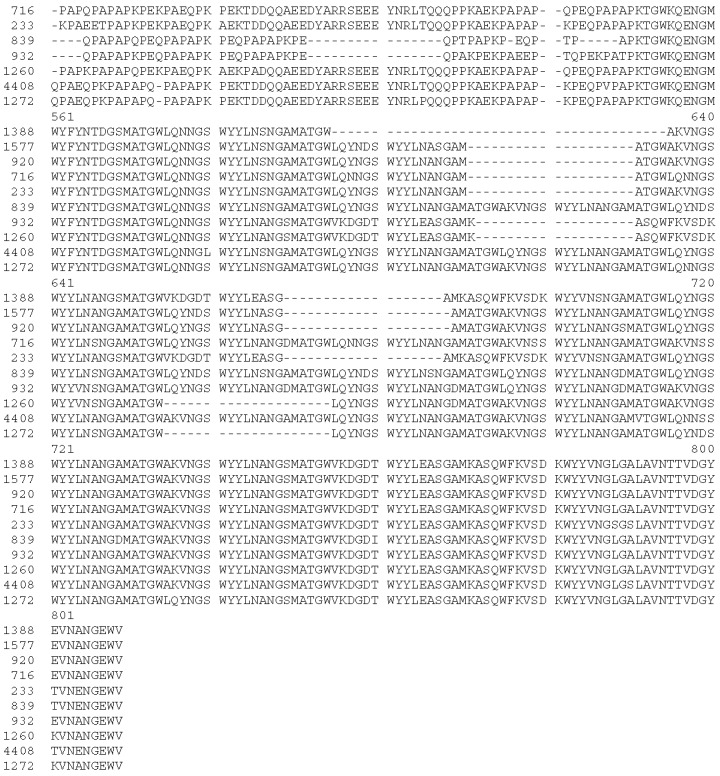
Alignment of deduced amino acid sequences of PspA of the tested *S. pneumoniae* clinical isolates. The alignment was performed with Multalin (http://multalin.toulouse.inra.fr/multalin/multalin.html). Gaps were inserted to give the best fit.

The breadth of cross-reactivity of the serum IgG was tested with nine additional clinical isolates expressing heterologous PspA proteins and different capsular serotypes in immunoblotting and flow-cytometric analyses. An alignment of deduced amino acid sequences of PspA of the tested clinical isolates is shown in Appendix Figure A3. Sera from CCL-NPB and CCL-PR1-immunized mice cross-reacted with all tested clinical isolates containing a NPB (Table 1). CCL-PR1 immune sera cross-reacted with nine out of the 10 tested strains. CCL-PR2/C and CCL-PR2/N induced IgG that cross-reacted with eight of the 10 genetically diverse pneumococcal clinical isolates tested. None of the pre-immune sera were positive in flow-cytometry or Western blot analyses.

### 3.5. Protective Capacity of Epitope-Loaded SVLPs

The capacity of recombinant proteins containing PRR regions to confer protection against pneumococcal challenge has been demonstrated previously [31]. In this study, a segment of PspA comprising the entire NPB+PR region from strain 1577 (rPspA) was cloned, produced in *E. coli* and tested in comparison with the synthetic epitope-loaded SVLPs in a challenge model of acute infection.

**Figure 5:**
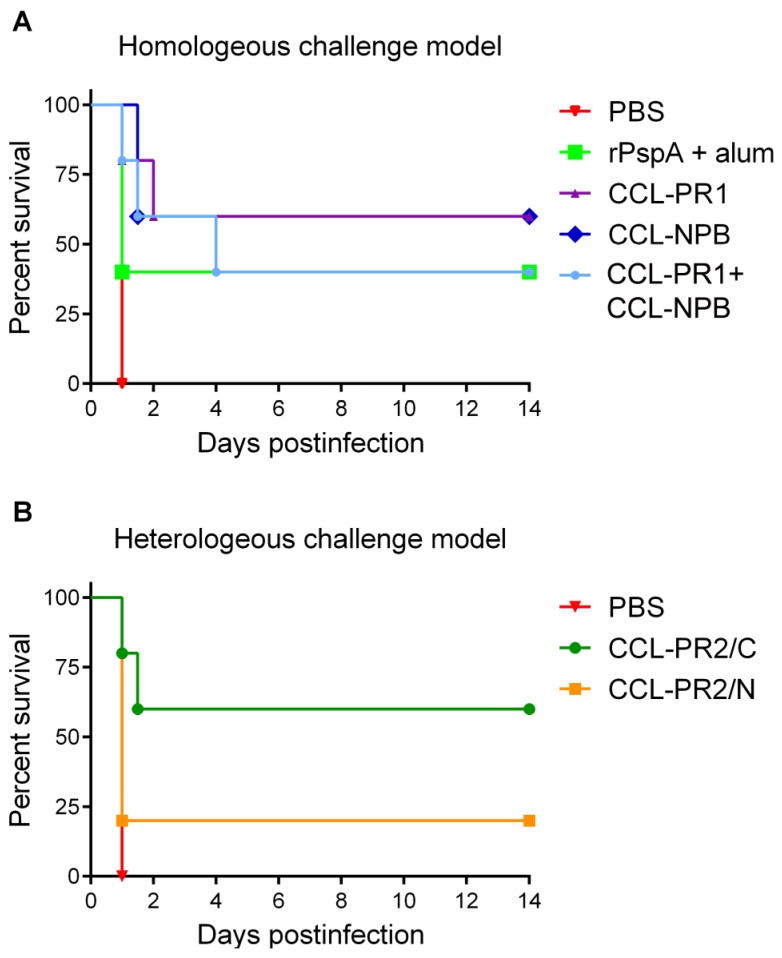
Evaluation of the protective efficacy of peptide-loaded SVLP vaccine candidates in an experimental mouse infection model. NMRI outbred mice were immunized with recombinant PspA (rPspA) formulated with alum or with SVLPs loaded with homologous (**A**) (CCL-PL1, CCL-NPB or mixture) or heterologous (**B**) (CCL-PR2/N or CCL-PR2/C) epitopes and infected intravenously (i.v.) with 10^4^ CFUs of serotype 1 *S. pneumoniae* 1577. Control mice received PBS prior to infection. The health status of the animals was monitored for 14 days post-infection. In (**A**), there was a statistically significant delay of time to morbidity of the CCL-PR1, CCL-NPB and CCL-NPB + CCL-PR1 combination groups compared to the PBS control group (*p* = 0.0143, *p* = 0.0027 and *p* = 0.0143, respectively) but no difference between the rPspA and the PBS group (*p* = 0.1336; log rank (Mantel-Cox) test; n = 5 mice per group). In (**B**), there was a statistically significant prolongation of survival for CCL-PR2/C immunized mice compared to PBS-treated animals (*p* = 0.0143) but no difference between the CCL-PR2/N and the PBS group (*p* = 0.3173; log rank (Mantel-Cox) test; n = 5 mice per group).

Mice were immunized either with rPspA formulated with alum, or with PRR mimetic-loaded SVLPs, and then challenged intravenously with a lethal dose of *S. pneumoniae* strain 1577. Mice that developed signs of illness (ruffled fur, hunched back, paralysis or other symptoms) were euthanized. Negative control mice that received PBS prior to challenge died or became moribund within 24 h after challenge (Figure 5A). Immunization with epitope-loaded SVLPs significantly delayed onset of illness and some mice had not developed disease symptoms by day 14 after the challenge, at which time the experiment was terminated. No difference in efficacy was observed between NPB and PR1-loaded SVLPs and both formulations were slightly more effective than rPspA. The combination CCL-NPB + CCL-PR1 (Figure 5A, mixture) elicited longer median survival time than rPspA but was comparable in terms of overall protection.

In order to determine the capacity of PRR epitope mimetic loaded SVLPs to confer protection against infection with pneumococci expressing a heterologous PRR, mice were immunized with SVLPs made from CCL-PR2/N and CCL-PR2/C and then challenged with 10^4^ CFUs of *S. pneumoniae* 1577. Immunization with CCL-PR2/N was only poorly protective against lethal infection, whereas CCL-PR2/C, in which the PRR-derived sequence is linked to the CCL in the reverse orientation, significantly increased the median survival time (Figure 5B). Some immunized mice did not die or show any symptoms of illness during the 14 day period of observation after infection.

### 3.6. Localization of Cross-Protective Epitopes in PR2

**Figure 6:**
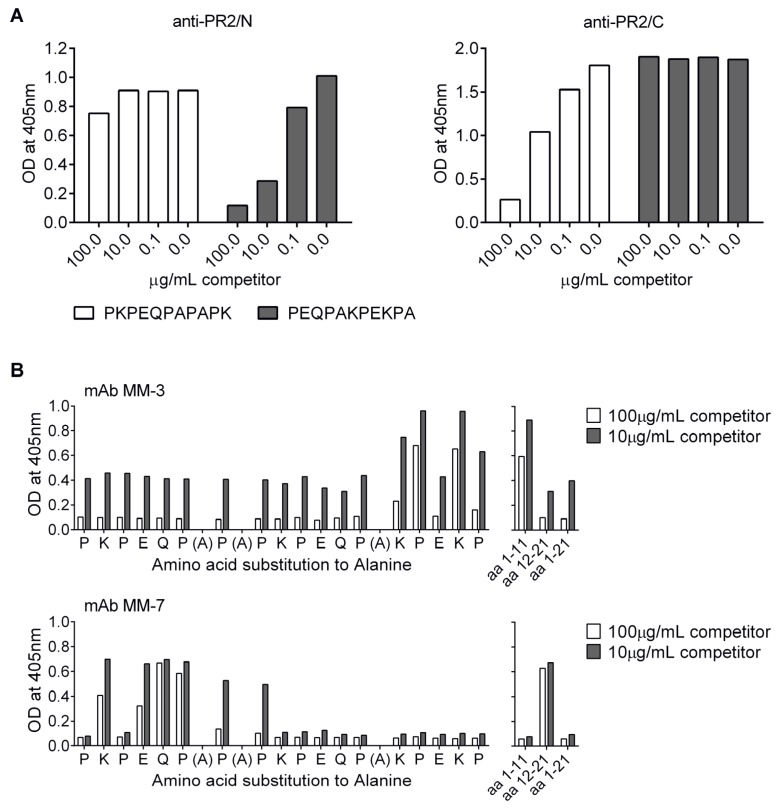

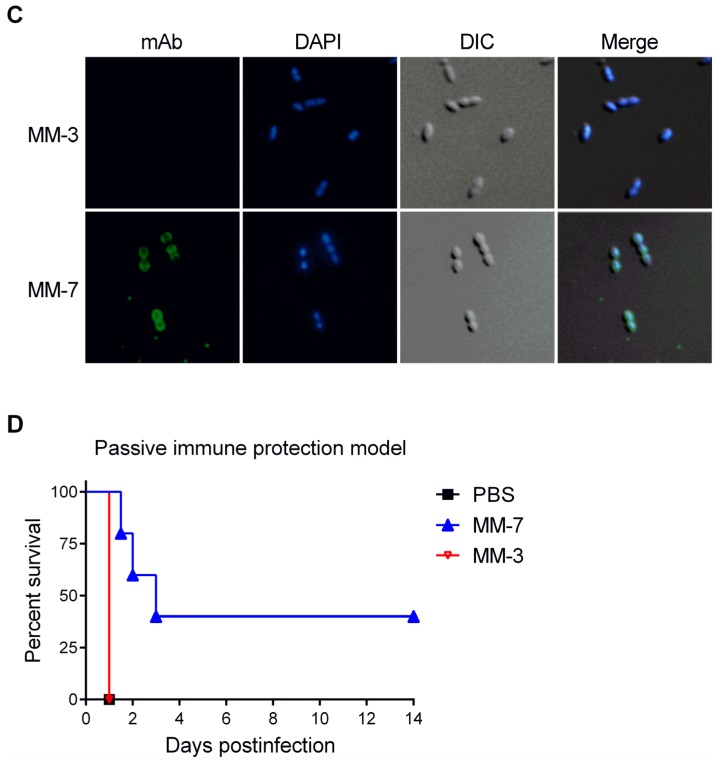
Localization of cross-protective epitopes in PR2. (**A**), Epitope mapping of anti-PR2/N and anti-PR2/C serum antibodies by competition ELISA with peptides representing N- and C-terminal parts of PR2. Immune sera were used at a dilution of 1:100 and the peptide competitors at a concentration of 100, 10 or 0.1 µg/mL; (**B**), Fine mapping of epitopes recognized by mAbs MM-3 and MM-7. MAbs were pre-incubated with peptides representing amino acids 1–11, 12–21 and 1–21 of the PR2-peptide, and an Ala-scan library of the PR2 sequence, at 10 or 100 µg/mL. Equilibrated antibody-competitor mixes were subsequently transferred to PR2-peptide coated ELISA plates. Bound antibody was detected using an alkaline phosphatase-conjugated secondary antibody; (**C**), Immunofluorescence staining of formalin-fixed *S. pneumoniae* 1557 with mAb MM-3 or MM-7. Surface bound IgG was detected with an Alexa Fluor 488-conjugated secondary antibody. Bacterial DNA was stained with 4,6-diamidino-2-phenylindole (DAPI). DIC, differential interference contrast image; (**D**), Mice were administered 0.5 mg of mAbs MM-3, MM-7 or PBS by i.v. injection. 12 h later mice were infected i.v. with 10^4^ CFUs of *S. pneumoniae* 1577. The health status of the animals was monitored and animals that developed signs of illness were euthanized, according to humane endpoints. There was a statistically significant delay of time to morbidity for MM-7-compared to PBS-treated mice (*p* = 0.0027) but no difference between the MM-3 mAb and the PBS group (*p* = 1; log rank (Mantel-Cox) test; n = 5 mice per group).

In order to map the epitopes conferring protection in the heterologous challenge experiment, competition ELISA was performed using anti-sera against CCL-PR2/N and CCL-PR2/C and peptide competitors representing the N- and C-terminal halves of the PR2 epitope. The results showed that the main fraction of the vaccine-induced IgG serum antibodies were directed against epitopes located at the unconjugated end of each peptide antigen; that is, against the C-terminal portion of CCL-PR2/N and the N-terminal segment of CCL-PR2/C (Figure 6A).

To characterize the epitopes in more detail, seven mAbs (MM-1 to MM-7) were generated from spleen cells taken from a CCL-PR2/N immunized mouse. The ability of the seven mAbs to bind PspA on streptococcal cells was analyzed by immunofluorescence microscopy. Only MM-7 cross-reacted with the native antigen and stained the bacterial cell surface (Figure 6C). The epitopes recognized by the mAbs MM-1 to MM-7 were mapped by competitive ELISA, using PR2-coated ELISA plates and competing peptides, including shortened peptides representing either amino acids 1–11 or 12–21 of PR2, as well as a panel of 18 synthetic 21-mer peptides representing an alanine scan of the PR2 sequence (Figure 6B). In this way, mAbs MM-1 to MM-6 were shown to bind to the C-terminal part and only the *S. pneumoniae* cross-reactive mAb MM-7 to the N-terminal part of PR2. Representative results for mAbs MM-3 and MM-7 are shown in Figure 6B. Moreover, the alanine-scan identified key residues in each motif that are important for binding to these two antibodies (see Figure 6B). These include KPEKP for binding MM-3, and KPEQP for recognition by MM-7.

### 3.7. Protection by Passive Transfer of Anti-PspA mAbs

Passive immunoprotection experiments were performed to test the protective efficacy of the seven mAbs against challenge with *S. pneumoniae* 1577. Purified mAbs formulated in PBS were administered to mice intravenously 12 h before infection with 10^4^ CFUs. Control mice received PBS 12 h prior to the challenge. Only mAb MM-7 was able to mediate partial protection against infection in this assay (Figure 6D). Hence, the N-terminal region of PR2 centered on the residues KPEQP was identified as a key protective epitope. It is notable that antibodies to this region of PR2 are elicited by CCL-PR2/C (Figure 6A), which also showed significant protective effects in the heterologous challenge model (Figure 5B). This motif is also present in the PR1 mimetic, which also showed significant protection in the homologous challenge study (Figure 5A).

## 4. Discussion

The main goal of this study was to explore the ability of SVLPs carrying synthetic epitopes from PspA to elicit protective humoral immune responses to a lethal *S. pneumoniae* infection in mice. Earlier studies had established that epitope-loaded SVLPs elicit very high titres of epitope-specific antibody responses without the need for co-administration of an adjuvant [19,20,22]. The B cell epitopes studied were derived from the circumsporozoite protein of the malaria parasite *Plasmodium falciparum* and from the gp120/gp41 surface spike on HIV-1. The adjuvant-independence of the strong pathogen-specific immune responses observed likely results from the properties of the SVLPs, which include multivalent surface display of B cell epitopes, multivalent T cell epitopes, as well as a lipidic group (the TLR2 ligand Pam_2_Cys) in the inner core of each nanoparticle. In this work, we focus on the bacterial pathogen *S. pneumoniae*, which provides an opportunity to address an important vaccine target pathogen and to exploit well-established *in vitro* and *in vivo* methods to demonstrate that a designed SVLP vaccine candidate can be protective in both active and passive mouse challenge experiments. This is an important step in exploring the potential of SVLPs for vaccine discovery and development.

PspA is a surface-associated choline-binding protein that is present in essentially all known clinical isolates. A bioinformatic analysis grouped known PspAs into three families and six clades; clades 1/2 in family one, clades 3/4/5 in family two, and clade 6 in family three [30]. PspA is immunogenic and despite numerous sequence variations remarkably cross-reactive between isolates. Most PspAs share many identical short stretches of amino acids, particularly in the PRR. Further cross-reactivity between pneumococci may also arise due to PRRs with similar short conserved motifs (such as PAPAP, PKP, or PEKP) present in the closely related surface virulence protein PspC [30,32,41].

PspA is attached to the pneumococcal surface through non-covalent interactions of its C-terminal choline-binding domain with teichoic acid or lipoteichoic acid in the cell membrane [42,43]. The PRR links the choline-binding domain with the N-terminal helical domain exposed on the pneumococcal surface, where it can bind and thereby block the membrane-disruptive antimicrobial activity of human lactoferrin [29,40]. Although the PRR threads through the thick peptidoglycan and capsule layers, at least portions of the PRR must be exposed and accessible to circulating antibodies [31]. The high proline content and frequent occurrence of repeated PXP and PXXP motifs suggests that this region might adopt extended polyproline-II conformations [44], an idea supported by circular dichroism studies of peptides from the PRR of the β-protein from group B streptococci [45]. The PRR region is therefore an attractive target for peptide vaccine design, using linear peptides, which will likely not be required to adopt a compact folded conformation. Hence, for our studies, linear peptides were used, derived from the NPB and the PRR of the challenge strain 1577 (NPB and PR1, Figure 1B), as well as the PRR from a heterologous strain (PR2).

All SVLP formulations were immunogenic in outbred mice and elicited IgG responses reactive with the synthetic peptides. Moreover, different peptides on separate SVLPs could be combined in one administration without interfering with individual anti-peptide antibody titers. The antisera contained antibodies binding to PspA in total lysates of the pneumococci in Western blotting analyses (Figure 4A), and stained cells upon flow cytometry analysis, showing that exposed epitopes are recognized on the surface of the bacteria. In both the homologous and heterologous challenge studies involving active immunizations, significant protection was observed for each of the peptide antigens tested (Figure 5). Moreover, in the homologous challenge experiment, under the conditions used here, the level of protection elicited by the SVLPs was at least as high if not higher than that using rPspA as immunogen with alum adjuvant (Figure 5A).

To further characterize epitopes mediating protection, spleen cells of one of the mice immunized with CCL-PR2/N were used to generate monoclonal antibodies. Of the seven mAbs produced (MM-1 to MM-7), only one (MM-7) cross-reacted with the native antigen by FACS analysis, and upon passive transfer and challenge with the bacterium, only MM-7 was able to confer partial protection in mice (Figure 6D). Interestingly, more detailed mapping studies with an Ala-scan library of peptides revealed that residues important for recognition by this mAb are located in a motif (PKPEQP) close to the N-terminus of the PR2 peptide (Figure 6B). Although Ab recognition of some epitopes may be influenced by the mode of peptide surface adsorption during ELISA, the results obtained are complementary to those reported by Daniels *et al.* [31], studying immune responses from rPspA fragments, who also identified the PKPEQ motif in the PRR as a mediator of protection against *S. pneumoniae*. However, the epitope mapping studies reported here also show (Figure 6A) that vaccine-induced IgG serum antibodies were directed mostly against epitopes located at the unconjugated end of the peptide antigens, *i.e.*, against the regions that should be more exposed on the surface of the nanoparticle. It seems likely, therefore, that antibodies directed towards this protective PKPEQP epitope might be more efficiently elicited by the PR2/C immunogen, than by PR2/N. It is also notable that this epitope is present near the N-terminus of the PR1 peptide immunogen, although likely not in an optimal location since again this peptide was N-conjugated to the CCL.

Since pneumococci are genetically highly diverse and capable of rapid recombinational changes [46], it is generally assumed that an effective pneumococcal subunit vaccine should incorporate multiple protective epitopes into a single vaccine formulation. Future efforts will therefore focus on identifying additional components that can be incorporated into an SVLP-based streptococcal vaccine candidate.

## 5. Conclusions

We have shown that linear epitopes derived from the proline-rich region of pneumococcal surface protein A can be incorporated in multiple copies onto the surface of self-adjuvanting synthetic lipopeptide-based nanoparticles (called here synthetic virus-like particles (SVLPs)). In this format, the epitopes are highly immunogenic and elicit strong humoral immune responses in mice without use of an additional adjuvant. The results further show that following active immunization and challenge with lethal doses of streptococcus, these SVLP-based immunogens elicit significant protection in mice, and therefore may have potential for the development of an effective synthetic vaccine against *Streptococcus pneumoniae*.

## Figures and Tables

**Figure A2:**
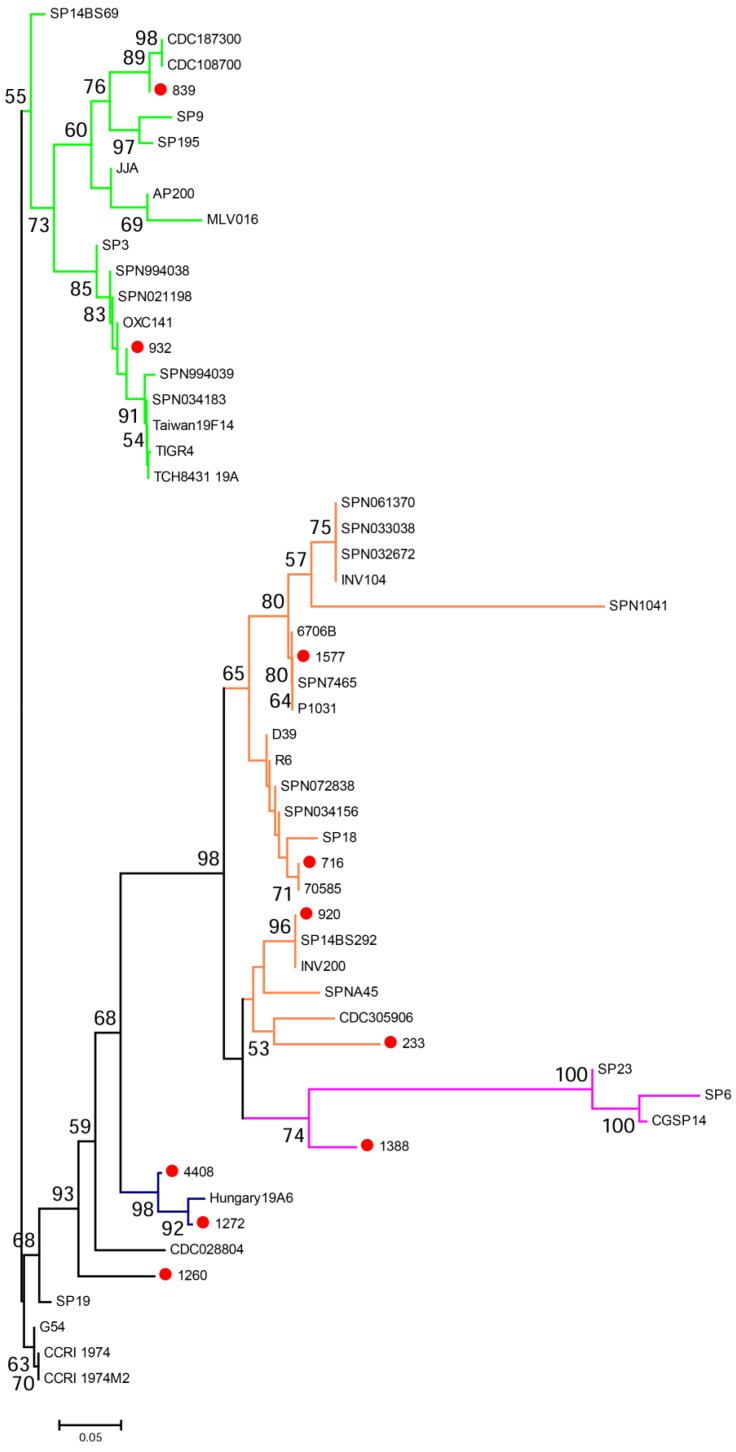
Maximum likelihood phylogenetic tree of the PRR sequences of 10 *S. pneumoniae* clinical isolates (marked with a red dot) and of 44 *S. pneumoniae* genomes from the NCBI database using a general time-reversible (GTR) substitution model with γ correction for among-site rate variation. The branches are annotated with their bootstrap support (numbers in the branch). Marked pneumococcal clinical isolates were tested for their cross-reactivity with serum IgG antibodies induced upon immunization with epitope-loaded SVLPs. Colored branches correspond to *pspA* allelic variants that have been classified into clades according to Hollingshead SK *et al.* [30], green: clade 3; orange: clade 1; pink: clade 2; blue: clade 5; black: clade 4).

## Appendix

**Table A1:** PCR primers used for sequencing *pspA* from clinical isolates (see Table 1).

Primer	Sequence
PspA1	GCAAAAGTAGTGTCTTTATCTTTTTGG
PspA2	CCAGCGTCGCTATCTTAGGGGCTGGTT
PspA3	CGAACATAAATCTATTCAATCGAACC
PspA4	AATCAACACCACCACTCATCC
PspA5	ACCACATACCGTTTTCTTGTTTCC
PspA6	TGGAAACAAGAAAACGGTATGTGG
PspA7	CGAACATAAATCTATTCAATCGAACC
PspA8	AGGTCGTGTGTGCTTCACG
PspA9	GGAAACAAGAAAACGGTATGTGGT
PspA10	TCAACAAGCTGAAGAAGACTATGC
PspA11	AATGAGTTAGGCCCTGATGG
PspA12	CGGCTTAAACCCATTCACC
PspA13	TCTCCATCTTTCACCCAACC
PspA14	AATGAGTTAGGCCCTGATGG
